# Association between Stable Coronary Artery Disease and In Vivo Thrombin Generation

**DOI:** 10.1155/2016/5149825

**Published:** 2016-08-11

**Authors:** Benjamin Valente-Acosta, Manuel Alfonso Baños-González, Marco Antonio Peña-Duque, Marco Antonio Martínez-Ríos, Leslie Quintanar-Trejo, Gad Aptilon-Duque, Mirthala Flores-García, David Cruz-Robles, Guillermo Cardoso-Saldaña, Aurora de la Peña-Díaz

**Affiliations:** ^1^Instituto Nacional de Cardiología Ignacio Chávez, Grupo Genética Intervencionista, Departamentos de Biología Molecular, Hemodinámica, Endocrinología, 14080 México City, Mexico; ^2^Departamento de Farmacología, Facultad de Medicina, Universidad Nacional Autónoma de México, 04510 México City, Mexico; ^3^División Académica de Ciencias de la Salud, Universidad Juárez Autónoma de Tabasco, Hospital Regional de Alta Especialidad “Dr. Juan Graham Casasús”, 86126 Villahermosa, TAB, Mexico

## Abstract

*Background*. Thrombin has been implicated as a key molecule in atherosclerotic progression. Clinical evidence shows that thrombin generation is enhanced in atherosclerosis, but its role as a risk factor for coronary atherosclerotic burden has not been proven in coronary artery disease (CAD) stable patients.* Objectives*. To evaluate the association between TAT levels and homocysteine levels and the presence of coronary artery disease diagnosed by coronary angiography in patients with stable CAD.* Methods and Results*. We included 95 stable patients admitted to the Haemodynamics Department, including 63 patients with significant CAD and 32 patients without. We measured the thrombin-antithrombin complex (TAT) and homocysteine concentrations in all the patients. The CAD patients exhibited higher concentrations of TAT (40.76 *μ*g/L versus 20.81 *μ*g/L, *p* = 0.002) and homocysteine (11.36 *μ*mol/L versus 8.81 *μ*mol/L, *p* < 0.01) compared to the patients without significant CAD. Specifically, in patients with CAD+ the level of TAT level was associated with the severity of CAD being 36.17 ± 24.48 *μ*g/L in the patients with bivascular obstruction and 42.77 ± 31.81 *μ*g/L in trivascular coronary obstruction, *p* = 0.002.* Conclusions*. The level of in vivo thrombin generation, quantified as TAT complexes, is associated with the presence and severity of CAD assessed by coronary angiography in stable CAD patients.

## 1. Introduction

Coronary artery disease (CAD) has been recognized as a chronic inflammatory disease. Proinflammatory cytokines and adhesion molecules play an important role in its initiation and progression [1, 2] by initiating a crosstalk between inflammation and the haemostatic system [3].

A key molecule in the haemostatic system is thrombin, a serine protease that primarily converts soluble fibrinogen into fibrin [4]. Thrombin is neutralized by its physiological inhibitor antithrombin; thus, the thrombin-antithrombin (TAT) complex is believed to be a reliable marker of in vivo thrombin generation [5].

Thrombin is a pleiotropic enzyme, performing various actions to activate protease-activated receptors expressed on the endothelial cells, leukocytes, vascular smooth-muscle cells, fibroblasts, and platelets. These actions result in multiple proatherogenic cellular responses including the enhancement of endothelial dysfunction and permeability, oxidative stress, apoptosis, and the overexpression of inflammatory cytokines that promote atherosclerotic plaque formation [6–8].

The importance of thrombin as a key promoter of atherosclerosis has only been shown in animal models [9, 10]. Bea et al. cleverly showed that a direct thrombin inhibitor could reduce the progression of atherosclerosis in apolipoprotein E-deficient mice through the inhibition of the transcription of multiple proinflammatory factors [9].

On the other hand, homocysteine (Hcy), an amino acid metabolized from methionine [11], enhances thrombin generation [12, 13]. High concentrations of homocysteine have been associated with an increased risk of atherosclerosis and arterial thrombosis [14].

In the clinical field, the evidence is inconsistent; some cross-sectional studies have shown a positive association between thrombin generation and aortic, carotid, and peripheral arterial atherosclerosis [15–17], whereas others have not [18–20].

In the present study, we aim to show the association between TAT levels as a marker for thrombin generation in vivo, homocysteine levels, and the presence and severity of coronary artery disease diagnosed by coronary angiography.

## 2. Material and Methods

### 2.1. Subjects

The study was performed in patients who were admitted to the Haemodynamics Department for a diagnostic coronary angiography because of stable chest pain, suspected for CAD. The study was carried out in the National Institute of Cardiology Ignacio Chavez.

The population included in our study consisted of 95 patients who underwent a coronary angiography, which was performed using femoral access in all patients. Patients were classified according to their coronary angiography as CAD+ when they had stenosis greater than 50% in at least one major coronary artery and as CAD− when they had no angiographic evidence of coronary occlusion.

The severity of coronary atherosclerosis was classified as one-, two-, or three-vessel disease according to the number of major coronary arteries that were stenotic.

We excluded patients who had undergone coronary bypass surgery or previous coronary intervention, those with infectious, neoplastic, or thyroid disease, kidney or liver failure, or a recent myocardial infarction, those who had been diagnosed with unstable angina within the last month, and those taking any type of anticoagulant.

For all the patients anthropometric measures and traditional risk factors were recorded. Individuals were considered to have diabetes mellitus type 2 if they had been previously diagnosed or were receiving hypoglycemic treatment and/or insulin. Individuals were considered to have hypertension if they had been previously diagnosed or were receiving an antihypertensive therapy. Dyslipidemia was defined as total cholesterol (TC) ≥ 200 mg/dL and/or low-density lipoprotein cholesterol (LDL-C) ≥ 130 mg/dL and/or triglycerides (TG) ≥ 150 mg/dL and/or high-density lipoprotein cholesterol (HDL-C) ≤ 40 mg/dL or by a previous diagnosis. Body mass index was calculated using a standard formula (weight (kg)/height (m)^2^).

### 2.2. Laboratory Measurements

From each patient, we obtained a blood sample after a fast of at least 8 hours and always before the angiography. Venous blood (10 mL) was drawn and placed in a tube with EDTA as an anticoagulant. The sample was then centrifuged at 5000 rpm for 15 minutes. The plasma was immediately distributed into aliquots and stored at −70°C for less than 6 months. The samples were analyzed in blocks to reduce interassay variability.

The TC and TG were measured by enzymatic methods (Roche-Syntex/Boehringer Mannheim, Germany). High-density cholesterol (HDL-C) was quantified after precipitating lipoproteins containing apolipoprotein B with phosphotungstate/Mg^2+^. Low-density cholesterol (LDL-C) was estimated using the modified Friedewald formula. Accuracy and precision of lipid measurements were under periodic surveillance by the Centers for Disease Control and Prevention service (Atlanta, GA).

The plasma concentrations of total Hcy (tHcy) were determined with a commercially available immunonephelometric assay (Dade Behring), and the values were expressed in *μ*mol/L. The TAT was quantified using an ELISA kit (Dade Behring), and the values were expressed in *μ*g/L.

The institutional ethics committee approved the protocol and informed consent was obtained from each participant.

### 2.3. Statistical Analysis

We used descriptive statistics expressed as numbers (percentages) in categorical variables whereas continuous variables were expressed as mean ± standard deviation (SD) and the median with interquartile range values in accordance with their distribution. Student's* t*-test or the Mann-Whitney test was performed to compare the differences between continuous variables according to their distribution. The Kolmogorov-Smirnov test was used as evidence of normality. Significant differences between the categorical variables were evaluated using the Chi square test. The Pearson coefficient was used to evaluate the correlation of plasma tHcy and TAT concentrations.

We performed multivariate analyses using logistic regression to calculate independent association of the presence and severity of CAD with levels of TAT, Hcy, and traditional risk factors. Also, we performed a receiver operating characteristics curve (ROC) to establish the sensitivity and specificity of TAT for CAD diagnosis. Statistical calculations were performed using SPSS, version 15.

## 3. Results

Significant CAD was detected in 63 patients who were classified as CAD+ of which 31 were subclassified as bivascular and 32 as trivascular, whereas 32 patients did not have significant stenotic lesions; hence they were classified as CAD−.

The clinical and biochemical characteristics of the patients are shown in Tables 1 and 2, respectively. Hypertension, smoking, dyslipidemia, and diabetes mellitus type 2 are more prevalent in the patients with CAD+ than in CAD− patients. There was no statistically significant difference in the body mass index (BMI) between patients. Moreover, there were no differences in triglyceride or HDL-C levels. However, the TC and LDL-C levels were higher in CAD− patients.

The thrombin-antithrombin complex concentration was higher depending on the severity of the coronary artery disease. In the CAD− group, the average concentration was 20.81 ± 13.59 *μ*g/L whereas it was 40.76 ± 29.47 in the CAD+ group (36.17 ± 24.48 *μ*g/L in the patients with bivascular coronary artery disease and 42.77 ± 31.81 *μ*g/L in the patients with a trivascular coronary obstruction, *p* = 0.002).

The concentration of total homocysteine was higher in the CAD+ group, at 11.36 ± 4.38, compared with 8.81 ± 3.72 in the CAD− group (*p* < 0.01). We found a positive correlation between the plasma concentrations of TAT and tHcy (*r* = 0.234, *p* = 0.022).

We analyzed the effect of high TAT concentrations on the risk of CAD in a multivariate logistic regression model which include adjustment for age, sex, diabetes mellitus type 2, hypertension, and dyslipidemia; a high TAT concentration increased the risk for CAD (OR 1.048 CI = 1.005–1.093, *p* = 0.027) as shown in Table 3. Conversely, tHcy was not related to a significant increase in risk (OR 1.123, CI = 0.933–1.352, *p* = 0.219).

**Figure 1:**
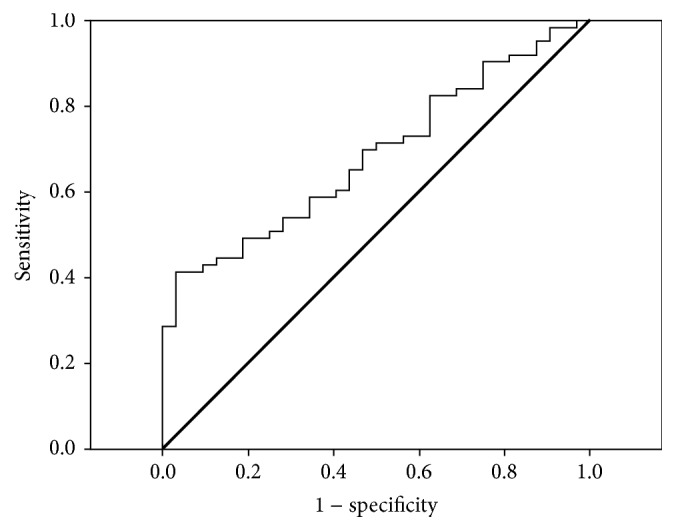
TAT sensitivity and specificity for CAD.

We used ROC curves to determine the sensitivity and specificity of serum TAT in patients with CAD. The value for TAT level to detect CAD patients with a sensitivity of 50% and specificity of 75% was 28.49 *μ*g/L. The area under the curve was 0.685 as shown in Figure 1.

## 4. Discussion

In our study, we investigated the relationship between thrombin generation and coronary artery disease diagnosed by coronary angiography. In our population of 95 Mexican patients with clinically suspected CAD, we were able to diagnose 63 patients with significant atherosclerotic lesions and 32 without significant CAD. We found that the concentration of TAT complexes, as a marker of in vivo thrombin formation, is related to the presence of CAD. Furthermore, TAT level was associated with the severity of the atherosclerotic burden in patients with stable but significant CAD. These findings are consistent with a previous study carried on by Borissoff et al. which provided evidence of a positive association between thrombin generation and the presence of CAD assessed by computed tomography [21].

The relationship between atherosclerosis and thrombin begins with a process mediated by PARs that triggers a multitude of phenotypic drifts leading to an endothelial dysfunction [7]. Additionally, thrombin has been shown to augment levels of mRNA that encode monocyte chemoattractant protein 1 (MCP-1) [22], a well-characterized chemokine abundant in human atherosclerotic plaques [23].

Furthermore, the transcription of IL-6, IL-8, and other inflammatory molecules is modified by thrombin, facilitating the recruitment of monocytes from the circulation into the arterial vessel wall [24]. Moreover, atherosclerosis positively correlates with an enhanced synthesis of reactive oxygen species (ROS), which tends to initiate multiple proatherogenic effects by facilitating lipid peroxidation and apoptotic processes [7]. This leads to the formation of an advanced plaque that has a low thrombomodulin concentration, allowing thrombin to augment the inflammatory stimuli [25].

We were also able to find a positive correlation between the TAT and homocysteine concentrations that agrees with previous reports showing a relationship between the degrees of coagulation activation, especially the generation of thrombin and the homocysteine concentration, in patients with acute coronary syndrome [18, 23]. This relationship could be explained by the inhibition of protein C activation and the downregulation of thrombomodulin by high concentrations of homocysteine [26].

It has been proposed that the treatment of hyperhomocysteinemia with an 8-week course of vitamins B_12_ and B_6 _could reduce the generation of thrombin [27]. This is important in our population because high concentrations of homocysteine can be caused by the thermolabile variant of the methylenetetrahydrofolate reductase whose frequency in the Mexican population is the highest worldwide [28].

New antithrombin agents, such as vorapaxar, have shown efficacy in reducing the risk of new cardiovascular events in secondary prevention strategy but also showed an increased hemorrhagic risk [29]; this raises new questions about whether patients with high baseline TAT benefited more of such therapy and compensate the hemorrhagic risk and the possible regression or attenuation of the atherosclerosis process [9, 30, 31].

Our study has some limitations, such as a small sample size enrolled in a single-center and a lack of patients with one-vessel disease, mostly due to the small sample size and the clinical characteristics of our population.

## 5. Conclusion

The level of thrombin generation, quantified as TAT complexes, is associated with the presence and severity of CAD assessed by coronary angiography in stable CAD patients.

## Figures and Tables

**Table 1:** Clinical characteristics of the CAD+ and CAD− groups.

Variables	CAD+	CAD−	*p*
	*n* = 63	*n* = 32	
Age (SD)	60.97 ± 9.97	47.91 ± 6.23	<0.01
Sex (M/F)	59/4	22/10	<0.01
BMI (kg/m^2^)	27.16 ± 3.61	28.2 ± 3.87	NS
DM2, *n* (%)	27 (42.9)	2 (6.3)	<0.01
Hypertension, *n* (%)	38 (60.3)	10 (31.3)	<0.01
Smokers, *n* (%)	11 (17.5)	5 (15.6)	NS
Dyslipidemia, *n* (%)	36 (57.1)	10 (31.3)	<0.05

The variables are expressed as the mean ± standard deviation (SD). A *t*-test was performed to compare the quantitative variables that exhibited a normal distribution; otherwise, a Mann-Whitney (nonparametric) test was performed. A Chi square distribution was calculated for the categorical variables. BMI = body mass index. DM2 = diabetes mellitus type 2.

**Table 2:** Biochemical characteristics of the CAD+ and CAD− groups.

Variables	CAD+	CAD−	*p*
	*n* = 63	*n* = 32	
Total cholesterol	151 (121–188)	170.21 (144.47–209.61)	<0.05
LDL-C	90.12 (61.04–115.08)	113.85 (88.7–152.58)	<0.01
HDL-C	33 (29–40)	33.32 (28.76–38.45)	NS
Triglycerides	151 (116–206)	135.34 (92.55–187.15)	NS
TAT	28.55 (15.53–60.12)	19.15 (9.23–29.48)	<0.01
tHcy	11.2 (8.52–13.3)	7.56 (6.73–9.87)	<0.01

The variables are expressed as the median and the interquartile range 25th–75th (IQR). A *t*-test was performed to compare the quantitative variables that exhibited a normal distribution; otherwise, a Mann-Whitney (nonparametric) test was performed.

**Table 3:** TAT concentrations on the risk of CAD after adjusting for traditional CAD factors using conditional logistic regression model.

Risk factor	*B* coefficient	Odds ratio (95% CI)	*p* value
Age	0.133	1.142 (1.05–1.24)	<0.01
Male sex	2.806	16.54 (0.928–294.81)	NS
Diabetes mellitus	3.700	40.43 (3.193–512.05)	<0.01
Hypertension	1.234	3.43 (0.757–15.58)	NS
Dyslipidemia	1.125	3.08 (0.718–13.20)	NS
Homocysteine	0.116	1.123 (0.933–1.35)	NS
*TAT*	*0.47*	*1.048 (1.005–1.09)*	*0.02*
